# Diagnostic Accuracy of [^68^Ga]Ga-PSMA-11 PET-CT in Characterising Bone Lesions in Prostate Cancer: A Single-Centre Study

**DOI:** 10.3390/cancers18071090

**Published:** 2026-03-27

**Authors:** Aishani Sachdeva, Mona Salem, John Jenkins, Kyle Wong, Gary J. R. Cook, Gurdip Azad

**Affiliations:** 1 Guys and St. Thomas NHS Trust, London SE1 7EH, UK; mona.salem2@nhs.net (M.S.); john.jenkins12@nhs.net (J.J.); wenkyle.wong@nhs.net (K.W.); gary.cook@kcl.ac.uk (G.J.R.C.); 2 Department of Clinical Oncology, Kasr Al-Ainy School of Medicine, Cairo University, Cairo 11956, Egypt; 3 The School of Biomedical Engineering and Imaging Sciences, Kings College London, St. Thomas Hospital, Westminster Bridge Road, London SE1 7EH, UK

**Keywords:** prostate cancer, PSMA PET, [^68^Ga]Ga-PSMA-11, bone metastasis, imaging accuracy, diagnostic performance

## Abstract

Background: Precise staging of prostate cancer is vital for treatment planning and prognosis. While [^68^Ga]Ga-PSMA-11 PET-CT has demonstrated high diagnostic accuracy in detecting metastatic disease, the interpretation of indeterminate or potentially benign PSMA-avid bone lesions remains a clinical challenge in routine practice. Methods: We conducted a retrospective single-centre study involving 214 patients who underwent [^68^Ga]Ga-PSMA-11 PET-CT between January 2021 and January 2024. Patients with prior known bone metastases or alternative PSMA radiotracers were excluded. Only those with follow-up imaging were included for diagnostic accuracy analysis. Follow-up modalities included PSMA PET-CT, CT, MRI, and bone scintigraphy. Final classification (metastatic or benign) was based on radiological and clinical assessment. Sensitivity, specificity, positive predictive value (PPV) and negative predictive value (NPV) were calculated using follow-up imaging as the reference standard. Lesions classified as indeterminate were analysed separately and excluded from diagnostic performance calculations. Results: Of the 214 included patients, 142 had follow-up imaging. Among 80 patients with bone lesions initially reported as metastatic, 74 (92.5%) were confirmed. Among 28 patients initially reported as having benign bone lesions, 26 (92.9%) remained benign on follow-up. Thirty-four patients with indeterminate lesions were reviewed; four were ultimately metastatic. Excluding indeterminate cases, sensitivity, specificity, PPV, and NPV were 97.4%, 86.7%, 94.9%, and 92.9%, respectively. Diagnostic discordance was primarily associated with benign uptake in the ribs, iliac bones, pubic rami and degenerative changes. Conclusions: [^68^Ga]Ga-PSMA-11 PET-CT shows excellent sensitivity and positive predictive value for detecting metastatic bone disease in prostate cancer. However, benign lesions may also exhibit uptake, emphasising the importance of integrating imaging results with PSA levels, Gleason scores, and TNM staging. Prospective studies are needed to validate these findings and assess their impact on long-term outcomes.

## Simple Summary

This retrospective single-centre study evaluated the diagnostic performance of [^68^Ga]Ga-PSMA-11 PET-CT for characterising bone lesions in patients with prostate cancer, with particular focus on the interpretation and outcomes of indeterminate lesions encountered in routine clinical practice. A total of 214 patients who underwent PSMA PET-CT between January 2021 and January 2024 were included, of whom 142 had follow-up imaging available to confirm lesion status. On initial imaging, bone lesions were classified as metastatic (40%), benign (39%), or indeterminate (21%) according to PSMA-RADS criteria. Follow-up imaging demonstrated that 74 of 80 lesions initially interpreted as metastatic were confirmed, while 26 of 28 benign lesions remained benign. Of the 34 indeterminate lesions, only 4 (11.8%) were subsequently confirmed as metastatic, with the majority representing benign findings. Excluding indeterminate lesions from diagnostic performance calculations, [^68^Ga]Ga-PSMA-11 PET-CT demonstrated a sensitivity of 97.4%, a specificity of 86.7%, a positive predictive value of 94.9%, and a negative predictive value of 92.9% for differentiating metastatic from benign bone lesions. These findings support the clinical utility of PSMA PET-CT for evaluating bone lesions in prostate cancer while highlighting the diagnostic uncertainty associated with indeterminate PSMA-avid lesions and the importance of cautious interpretation.

## 1. Introduction

Prostate cancer is a commonly diagnosed malignancy among men and the 2nd leading cause of cancer-related deaths in men in the UK [1]. Accurate staging of prostate cancer is essential for determining appropriate treatment strategies, particularly in identifying patients with metastatic disease, which influences both prognosis and treatment intent.

Recent advances in the treatment of metastatic prostate cancer suggests that patients with oligometastases can be treated more radically, depending on the location and number of lesions [2]. It has been shown that, in patients with high-risk or biochemically recurrent prostate cancer, prostate-specific membrane antigen (PSMA) positron emission tomography computed tomography (PET-CT) offers superior diagnostic accuracy over conventional imaging modalities like computed tomography (CT) and bone scintigraphy [3,4,5], leading to changes in clinical management [6,7].

However, the specificity of PSMA PET-CT, particularly with respect to bone lesions, remains a limitation. Benign bone conditions such as degenerative joint disease, fibrous dysplasia, or haemangiomas may exhibit increased PSMA uptake, leading to false positives [8,9,10]. Ribs are a recognised area of false positive findings with most PSMA tracers [11,12]. Differences between radiotracers (e.g., [^18^F]PSMA-1007 vs. [^68^Ga]Ga-PSMA-11) further complicate the diagnostic outcome, with the former associated with a higher incidence of equivocal bone lesions [13,14,15,16].

While the diagnostic performance of PSMA PET-CT for detecting metastatic prostate cancer has been widely reported, less attention has been given to the clinical interpretation and outcomes of indeterminate or potentially benign bone lesions detected on PSMA PET imaging [17]. In routine clinical practice, these lesions frequently present diagnostic uncertainty and may influence treatment decisions. Understanding how such lesions evolve on follow-up imaging is therefore important for improving diagnostic confidence and avoiding misclassification of benign findings as metastatic disease.

In this study, we aim to evaluate the accuracy of [^68^Ga]Ga-PSMA-11 PET-CT in characterising bone lesions using real-world data from a single institution, with particular focus on the clinical outcomes of metastatic, benign, and indeterminate lesions following follow-up imaging. Rather than focusing solely on diagnostic accuracy, our study explores how initial imaging impressions evolve on follow-up imaging, which may influence clinical decision making and patient management. It also provides insight into the practical interpretation challenges encountered by reporting radiologists and nuclear medicine physicians.

## 2. Materials and Methods

This retrospective, single-centre study was conducted at King’s College London and Guy’s and St Thomas’s NHS Foundation Trust. The study was approved by the institutional Cancer Cohort Ethics Committee (REC Reference 23/NW/0105), IRAS Project ID 325735.

### 2.1. Patient Selection

All patients who underwent a PSMA PET-CT scan between January 2021 and January 2024 were identified through the Picture Archiving and Communication System (PACS) IDS7 Version 26.2, using the following search terms:

- “Exam Description” containing the keyword “*PSMA*”.
- “Examination Date” from 1 January 2021 onwards.

Scans reporting bone involvement were reviewed in detail. The following inclusion and exclusion criteria were applied:

Inclusion criteria:

- Patients who underwent PET-CT using the [^68^Ga]Ga-PSMA-11 radiotracer.
- PSMA PET-CT performed for:
  (a) Initial staging of high-risk prostate cancer.
  (b) Evaluation of biochemical recurrence.
- Availability of follow-up imaging for lesion verification.
  Exclusion criteria:
- Use of any PSMA radiotracer other than [^68^Ga]Ga-PSMA-11.
- Known bone metastases prior to the PSMA PET-CT scan.
- Absence of follow-up imaging.

### 2.2. Reference Standard

Follow-up imaging was used as the reference standard for characterising bone lesions initially detected on [^68^Ga]Ga-PSMA-11 PET-CT. This included:

- Repeat PSMA PET-CT scans of any type.
- Conventional CT scans.
- MRI scans.
- Bone scintigraphy.

### 2.3. Imaging Interpretation

Follow-up scans, reported by experienced radiologists, were considered the “ground truth” for determining the final classification of bone lesions as:

- Benign.
- Metastatic.
- Indeterminate.

All PSMA PET-CT scans were interpreted as part of routine clinical reporting by consultant nuclear medicine physicians and radiologists with experience in oncologic PET imaging at our institution. Lesions were categorised using the PSMA-RADS 2.0 classification framework [18,19] where appropriate. As this was a retrospective study based on clinical reports retrieved from the PACS, formal inter-reader agreement analysis was not performed.

Metastatic lesions either had typical morphological appearances of metastasis, showed response to treatment, including increased or appearance of sclerosis, or showed progressive changes typical of metastatic disease. Benign lesions typically did not have these features, and indeterminate lesions did not fit into either of these groups.

### 2.4. Data Collection

Additional clinical data were collected from patient records, including:

- Prostate-specific antigen (PSA) level at presentation.
- Gleason score.
- TNM staging.

Radiological interpretations and clinical data were analysed to seek any correlations.

### 2.5. Statistical Analysis

Descriptive statistics were used to characterise the study population. Sensitivity, specificity, positive predictive value (PPV), and negative predictive value (NPV) of [^68^Ga]Ga-PSMA-11 PET-CT for diagnosing metastatic bone lesions were calculated using standard formulas. Then, 95% confidence intervals were calculated using the Wilson’s score due to the small sample size.

Lesions initially classified as indeterminate on [^68^Ga]Ga-PSMA-11 PET-CT were excluded from diagnostic performance calculations, as they could not be definitively categorised as positive or negative findings at the time of initial interpretation. These lesions were analysed separately to assess their final classification on follow-up imaging. Data analysis was performed using Microsoft Excel, Version 16.100.4 (25090553).

## 3. Results

**Figure 1:**
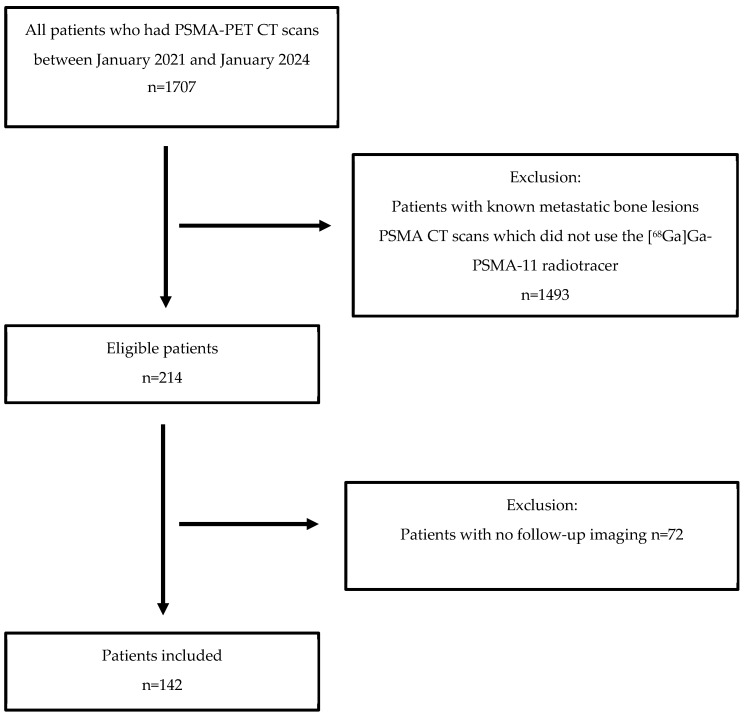
Patient flow diagram depicting inclusion and exclusion criteria.

A total of 1707 patients underwent [^68^Ga] Ga-PSMA-11 PET-CT scans between January 2021 and January 2024. Of these, 214 patients met our inclusion criteria and were included in the final analysis (Figure 1). Among the included patients, 133 underwent PSMA PET-CT scans for initial staging of high-risk prostate cancer, and 81 were scanned due to suspected biochemical recurrence (Table 1).

The median duration of follow-up was 802 days (range: 5–1319 days); this was calculated from the date of initial PET-CT to last clinical follow-up. On reviewing the available follow-up records, none of the patients with benign or indeterminate lesions re-presented with new metastatic bone disease during the follow-up period of this study. Five patients could not be included in this analysis as they continued follow-up solely under urology and follow-up information was not available.

Based on the initial [^68^Ga]Ga-PSMA-11 PET-CT interpretation, of the 214 patients, 85 (40%) were reported to have metastatic, 46 (21%) indeterminate and 83 (39%) benign bone lesions. Only 142 (66.3%) patients had follow-up scans. Of the remaining 72 (33.6%) who did not undergo follow-up imaging, 55 had benign bone lesions interpreted on initial [^68^Ga]-Ga-PSMA-11 PET-CT, 5 had metastatic lesions and were treated with systemic therapy and 12 had indeterminate lesions.

**Figure 2:**
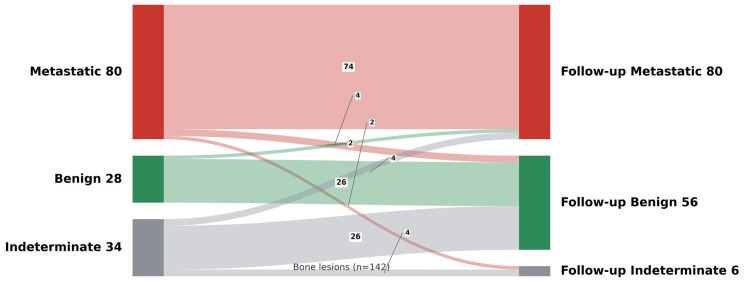
Sankey diagram showing reclassification of 142 bone lesions from initial PSMA PET impression to follow-up imaging characterisation. Flows represent the number of lesions categorised as metastatic, benign, or indeterminate on the initial PSMA PET scan and their corresponding classification on follow-up imaging.

Among the 142 (66%) patients with available follow-up imaging, 80 (56%) were found to have metastatic, 28 (20%) benign and 34 (24%) indeterminate lesions on the initial [^68^Ga]Ga-PSMA-11 PET-CT (Table 2, Figure 2).

Of the 80 patients initially reported to have metastatic bone lesions, 74 (92.5%) were confirmed on follow-up imaging. In six (7.5%) patients, follow-up suggested either benign (n = 4) or indeterminate (n = 2) lesions, indicating possible false positives (Table 2, Figure 2).

Among the 34 patients with initially reported indeterminate bone lesions (Table 2, Figure 2), 4 (11.8%) were later confirmed as metastatic, 26 (76.5%) benign and 4 (11.8%) remained indeterminate. This finding suggests that most indeterminate PSMA-avid bone lesions in our cohort were ultimately benign, highlighting the importance of cautious interpretation and follow-up imaging before classifying these lesions as metastatic disease.

Most benign lesions demonstrated imaging features consistent with non-malignant aetiologies such as fibrous dysplasia, haemangioma, or degenerative skeletal changes. This finding is clinically important because indeterminate PSMA-avid bone lesions represent a frequent diagnostic dilemma in clinical practice.

Among 28 patients with initially reported benign lesions, 2 patients (7.1%) were later found to have metastatic lesions, suggesting false-negative initial interpretations. In total, 26 (92.9%) patients were confirmed to have benign lesions on follow-up imaging (Table 2).

Overall, of the 142 patients with follow-up imaging, 36 patients (25.3%) had a change in their initial bone disease classification (Table 2).

The diagnostic performance of [^68^Ga]Ga-PSMA-11 PET-CT for differentiating metastatic from benign bone lesions was as follows: sensitivity 97.4% (95% CI 90.9–99.3%), specificity 86.7% (95% CI 70.3–94.7%), PPV 94.9% (95% CI 87.5–98.0%), NPV 92.9% (95% CI 77.4–98.0%). The diagnostic accuracy was 94.3% (95% CI 88.2–97.4%) (Table 3).

In the 80 patients with confirmed metastatic bone lesions, there was some correlation between metastases and Gleason score, but this was not statistically significant. In total, 36 (45.0%) patients had Gleason 8–10, 26 (32.5%) had Gleason 7 (4 + 3), 12 (15.0%) had Gleason 3 + 4, 3 (3.8%) had Gleason 6, and 3 (3.8%) had no Gleason scores available (Table 1). Interestingly, 17 patients with Gleason scores (8–10) had lesions initially reported as benign. These patients typically exhibited lower uptake and showed no progression on follow-up scans, highlighting that high Gleason score alone does not guarantee bone metastasis. Correlation with clinical features and follow-up imaging remains essential to avoid misclassification.

There was also some correlation between metastases and PSA levels, but this was not statistically significant. In total, 35 (43.8%) patients had PSA ≥ 20, 18 (22.5%) had PSA 10–20, 23 (28.8%) had PSA < 10, and 4 (5.0%) had missing PSA data (Table 1).

With respect to TNM staging, 34 patients had localised T1–T2N0M0 disease. A further 51 patients had more advanced disease with T3 or higher stage, without evidence of nodal or metastatic disease. Seven patients had nodal involvement (N1) at diagnosis, irrespective of T stage, while 38 patients presented with M1 disease, indicating de novo metastatic prostate cancer with bone involvement. TNM staging information was unavailable for 12 patients.

## 4. Discussion

In this retrospective single-centre study, we evaluated the diagnostic performance of [^68^Ga]Ga-PSMA-11 PET-CT for characterising bone lesions in patients with prostate cancer using real-world clinical data. Our findings demonstrate that [^68^Ga]Ga-PSMA-11 PET-CT showed high sensitivity and positive predictive value for distinguishing metastatic from benign bone lesions within this cohort. We found a sensitivity of 97.4% and a specificity of 86.7%, highlighting the modality’s strength in detecting true metastatic disease, with a relatively low false-negative rate.

Our results are broadly consistent with previously published studies reporting high sensitivity (97.4%) of [^68^Ga]Ga-PSMA-11 PET-CT for detecting bone metastases in prostate cancer [20,21,22,23,24]. However, in our cohort, specificity observed was somewhat lower than that reported in some prior studies. This difference may reflect several factors, including variations in patient populations, lesion classification criteria, and the use of follow-up imaging rather than histopathology as the reference standard to confirm lesion status. In contrast to studies using histopathological confirmation, our analysis relied on longitudinal imaging follow-up which is more representative of routine clinical practice but may influence estimates of diagnostic accuracy. Additionally, our study included lesions initially reported as benign or indeterminate in routine reporting, which may contribute to differences in specificity between cohorts. Our study found a positive predictive value of 94.9% and negative predictive value of 92.9%, confirming the reliability of [^68^Ga]Ga-PSMA-11 PET-CT in the diagnostic workup of prostate cancer patients at risk of metastatic bone involvement.

A key observation in this study relates to the behaviour of indeterminate bone lesions identified on PSMA PET-CT. In routine clinical practice, such lesions often present a diagnostic challenge and may influence treatment decisions. In our cohort, only 11.8% of lesions initially classified as indeterminate were ultimately confirmed as metastatic on follow-up imaging, while the majority were benign. These findings suggest that indeterminate PSMA-avid bone lesions frequently represent benign processes and highlight the importance of cautious interpretation and correlation with clinical parameters before classifying these findings as metastatic disease. This observation may help improve diagnostic confidence and reduce the risk of overtreatment based solely on equivocal PSMA PET-CT findings.

Among patients initially reported as having metastatic bone lesions, 7.5% were later determined to be either benign or indeterminate, a key limitation of PSMA PET-CT. Other studies, such as Pattison et al. and Dias et al., have also reported challenges distinguishing true metastases from benign uptake, particularly with certain PSMA radiotracers [14,16].

Indeterminate lesions accounted for nearly a quarter (24%) of our study cohort at baseline. Only a small fraction (11.8%) of these were later confirmed metastatic, while most were benign or remained inconclusive. This highlights the importance of integrating lesion morphology, anatomical location, and tracer uptake pattern, as well as considering a period of surveillance or MRI correlation rather than initiating immediate treatment as per PSMA-RADS [18,19].

The distribution of Gleason scores within our cohort also showed that metastatic bone disease was more frequently seen in patients with higher-grade tumours. Specifically, in patients with confirmed metastatic lesions, 45% had Gleason scores of 8–10 and 32.5% had Gleason 7 (4 + 3) scores. These observations are consistent with previous studies suggesting that clinical parameters such as PSA level, Gleason score, and imaging characteristics may be associated with metastatic disease detected on PSMA PET imaging, although such relationships require larger cohorts and dedicated analyses to establish independent predictive value [25]. In our cohort, this observation reflects the distribution of tumour grade among patients with metastatic disease within this cohort rather than representing a predictive measure of metastasis.

In contrast, PSA levels were a less reliable predictor, with nearly one third of metastatic patients presenting with PSA < 10 ng/mL. These findings align with prior research emphasising the added value of clinical risk stratification alongside imaging interpretation [26]. Though there was some association between metastatic disease and PSA level, the study was not powered to detect statistically significant correlations or evaluate predictive associations.

From a clinical perspective, these findings have implications for patient management [27]. In the era of PSMA PET-CT-guided treatment strategies, imaging findings can directly influence decisions regarding systemic therapy, local treatment, or metastasis-directed therapy [28]. Our results indicate that there are pitfalls in the interpretation of PSMA-avid lesions [29], especially indeterminate PSMA-avid bone lesions which may not necessarily represent metastatic disease and therefore may not always warrant immediate escalation of treatment. Instead, careful clinical correlation and short-interval follow-up imaging may be an appropriate strategy in selected patients, particularly when the lesion morphology or tracer uptake is atypical for metastatic disease. Such an approach may help reduce the risk of overtreatment while maintaining vigilance for true metastatic progression.

Emerging imaging approaches such as dynamic PSMA PET acquisition have also been proposed to improve lesion characterisation [30]. By analysing tracer kinetics over time, dynamic imaging may help differentiate malignant lesions from benign processes that demonstrate lower or delayed tracer uptake. Although dynamic PET imaging was not performed in the present study, this technique may offer additional diagnostic information in cases with equivocal PSMA uptake and warrants further investigation in prospective studies.

We acknowledge several limitations of this study. First, this was a retrospective analysis conducted at a single tertiary referral centre, with institution-specific imaging protocols, reader expertise, and follow-up practices. As such, the findings may not be fully generalisable to other institutions or patient populations, variable follow-up imaging intervals, and lack of histopathological confirmation of the bone metastases. Second, follow-up imaging, rather than histopathology, was used as the reference standard for confirming lesion status. Follow-up investigations included a variety of imaging modalities such as PSMA PET-CT, CT, MRI and bone scintigraphy, reflecting routine clinical practice but introducing verification bias, as not all lesions underwent assessment with the same modality. However, follow-up imaging interpretation was not independent of the baseline PSMA PET-CT findings, as radiologists had access to prior imaging during routine clinical reporting. These factors may influence estimates of diagnostic performance and represent an inherent limitation of retrospective imaging studies. Nevertheless, the use of longitudinal imaging follow-up allowed the assessment of lesion behaviour over time and provides clinically relevant insight into the real-world interpretation of PSMA PET-CT findings.

Another limitation related to the handling of indeterminate lesions is as follows. Lesions classified as indeterminate on initial PSMA PET-CT were excluded from the calculation of sensitivity and specificity because they could not be definitively categorised as positive or negative findings. While this approach is commonly used in diagnostic accuracy studies, it may lead to an overestimation of diagnostic performance, particularly in clinical settings where indeterminate PSMA PET findings are relatively common. Nevertheless, we analysed these lesions separately to provide additional clinical context regarding their behaviour on follow-up imaging.

Finally, the statistical analysis in this study was primarily descriptive, reflecting the diagnostic accuracy focus of the study. Associations between metastatic disease and clinical variables such as PSA level and Gleason score were exploratory and the study was not powered to detect statistically significant correlations or perform multivariable modelling. Larger prospective studies would be required to evaluate independent predictors of metastatic bone disease using PSMA PET-CT imaging.

Despite these limitations, the present study provides insight into the real-world interpretation of PSMA PET-CT bone findings, particularly regarding indeterminate lesions. The results highlight the importance of integrating imaging findings with clinical context and follow-up evaluation when interpreting PSMA-avid bone lesions in patients with prostate cancer.

Prospective studies with standardised follow-up protocols and histological validation are warranted to reduce uncertainty in interpreting indeterminate lesions, clarify the added value of PSMA PET-CT in long-term outcomes, and explore other strategies such as dual-tracer imaging or machine learning-based lesion classification.

## 5. Conclusions

Our study demonstrates that [^68^Ga]Ga-PSMA-11 PET-CT demonstrates high sensitivity and positive predictive value in characterising bone lesions in this cohort of patients. These findings confirm its value in staging and restaging in high-risk and biochemically recurrent prostate cancer. However, false positives, primarily involving benign skeletal conditions, highlight the need for cautious interpretation, particularly when imaging findings are discordant with clinical parameters.

The frequent occurrence of indeterminate lesions, most of which were ultimately benign, suggests that conservative management strategies including short-interval follow-up may be preferable to immediate systemic therapy. The integration of clinical features, especially Gleason score and TNM stage, can improve diagnostic confidence and reduce misclassification.

This study provides real-world evidence supporting the clinical utility of [^68^Ga]Ga-PSMA-11 PET-CT in characterising bone lesions in prostate cancer. However, the limitations of retrospective design, variable follow-up imaging, and lack of histopathologic confirmation warrant prospective studies to further refine diagnostic pathways and evaluate whether improved lesion characterisation leads to better patient outcomes.

## Figures and Tables

**Table 1:** Summary of patient characteristics included in study including indication for PSMA imaging and correlation between Gleason and PSA scores with confirmed metastatic disease.

Patient Characteristics		
	**Category**	**Number of Patients**
Indication for imaging	High-risk	133
	Biochemical recurrence	81
		**Number of Patients with Confirmed Metastatic Disease**
Gleason Score	8–10	36 (45%)
	4 + 3 = 7	26 (32.5%)
	3 + 4 = 7	12 (15%)
	6	3 (3.8%)
PSA level	>20	35 (43.8%)
	10–20	18 (22.5%)
	<10	23 (28.8%)

**Table 2:** Summary table illustrating the initial PSMA PET scan impression, how many of the lesions were confirmed according to their original classification and how many changed from their original impression.

	Initial PSMA PET Scan Classification (n, % Classification)	Confirmed Malignancy on Follow-Up Imaging (n, % of Each Group)	Confirmed Benign on Follow-Up Imaging (n, % of Each Group)	Indeterminate on Follow-Up Imaging (n, % of Each Group)	Classification Changed from Original Impression (n, % of Each Group)
Metastatic	80 (94%)	74 (92.5%) True positive	4 (5%) False positive	2 (2.5%)	4 (2.8%) (to benign)
Benign	28 (33.7%)	2 (7.1%) False negative	26 (92.9%) True negative	0 (0%)	2 (1.4%) (to malignant)
Indeterminate	34 (74%)	4 (11.7%)	26 (76.5%)	4 (11.7%)	30 (22.1%) (reclassified)
Total	142	80	56	6	36 (25.3%)

**Table 3:** Summary table of sensitivity, specificity, PPV, NPV and accuracy data.

	Sensitivity	Specificity	PPV	NPV	Diagnostic Accuracy
%, 95% CI	97.4 [90.9–99.3]	86.7 [70.3–94.7]	94.9 [87.5–98.0]	92.9 [77.4–98.0]	94.3 [88.2–97.4]

## Data Availability

The data presented in this study are available on request from the corresponding author due to patient details.
